# Overproduction, Purification, and Stability of the Functionally Active Human Carnitine Acetyl Transferase

**DOI:** 10.1007/s12033-022-00522-z

**Published:** 2022-06-21

**Authors:** Deborah Giudice, Lara Console, Arduino Arduini, Cesare Indiveri

**Affiliations:** 1 Department of Biology, Ecology and Earth Sciences (DiBEST), Laboratory of Biochemistry, Molecular Biotechnology and Molecular Biology, University of Calabria, Via P. Bucci 4c, 87036 Arcavacata di Rende, Italy; 2 Unical Cure S.R.L, Via P. Bucci 4d, 87036 Arcavacata di Rende, Italy; 3 Institute of Biomembranes, Bioenergetics and Molecular Biotechnology (IBIOM), National Research Council (CNR), Via Amendola 122/O, 70126 Bari, Italy

**Keywords:** Transferase enzyme, Mitochondria, Drug design, Carnitine, Metabolism

## Abstract

Human Carnitine Acetyl Transferase (hCAT) reversibly catalyzes the transfer of the acetyl-moiety from acetyl-CoA to L-carnitine, modulating the acetyl-CoA/CoA ratio in mitochondria. Derangement of acetyl-CoA/CoA ratio leads to metabolic alterations that could result in the onset or worsening of pathological states. Due to the importance of CAT as a pharmacological target and to the European directive for reducing animal experimentation, we have pointed out a procedure to produce a recombinant, pure, and functional hCAT using the *E. coli* expression system. The cDNA encoding for the hCAT was cloned into the pH6EX3 vector. This construct was used to transform the *E. coli* Rosetta strain. The optimal conditions for the overexpression of the fully active hCAT include induction with a low concentration of IPTG (0.01 mM) and a low growth temperature (25 °C). The recombinant protein was purified from bacterial homogenate by affinity chromatography. The pure hCAT is very stable in an aqueous solution, retaining full activity for at least two months if stored at − 20 °C. These results could be helpful for a broad set of functional studies on hCAT, including drug-design applications.

**Supplementary Information:**

The online version contains supplementary material available at 10.1007/s12033-022-00522-z.

## Introduction

Human Carnitine Acetyl Transferase (hCAT) is a monomeric enzyme of 626 amino acid residues, including two identical, tightly interacting domains. A solvent-accessible open cavity (tunnel) is present in the middle of the protein structure, which constitutes the substrate-binding site [1]. The 3D structure of hCAT is available. It shows a subtle shift of the side chains of some residues in the L-carnitine binding site, but no significant structural changes occur on substrate binding [1]. This enzyme catalyzes an equilibrium reaction that transfers the acetyl-moiety of acetyl-CoA to L-carnitine or *vice-versa* [2]. This reaction is crucial in buffering the excess acetyl groups and guaranteeing a fine-tuning of the acetyl-CoA/free CoA ratio in mitochondria. Indeed, although the amount of free CoA in mammalian cells is relatively small, it is engaged in several key metabolic pathways [3–5]. Its ester derivative, acetyl-CoA, is one of the most prominent allosteric modulators of many cellular processes, including epigenetic mechanisms [6]. In particular, it is an efficient activator of pyruvate dehydrogenase kinase (PDHK) that, in turn, inhibits pyruvate dehydrogenase (PDH), limiting the flux of glycolytic pyruvate into the tricarboxylic acid cycle (TCA). CAT may efficiently decrease acetyl-CoA concentration by pulling the equilibrium toward acetyl-carnitine, thus releasing free CoA. This process will result in activation of PDH, improving aerobic pyruvate oxidation with beneficial effects on energy metabolism [5, 7]. Acetyl-CoA modulates metabolic fluxes by allosteric activation of pyruvate carboxylase, a key enzyme in hepatic gluconeogenesis. The activity of this enzyme is essential for conveying pyruvate coming from extrahepatic lactate or alanine into gluconeogenesis. This pathway, which is important in providing glucose to neurons and erythrocytes during fasting, plays a major role in establishing the hyperglycaemic condition of diabetic patients [5]. Indeed, type 2 diabetes is characterized by high rates of hepatic glucose production through gluconeogenesis. In this condition, an increase of the acetyl-CoA pool in liver mitochondria will cause an increase in gluconeogenetic flux, affecting glucose utilization [8]. The high level of acetyl-CoA in mitochondria also exacerbates mitochondrial protein acetylation, a reaction that occurs by a non-enzymatic mechanism. CAT deficiency causes an increase in mitochondrial acetylproteome. Therefore, CAT also plays the role of modulating protein acetylation [9], which causes various changes in the activity of several mitochondrial proteins such as the acyl-CoA dehydrogenase, the citrate carrier [10], and the carnitine carrier [11]. Interestingly the carnitine carrier is also involved in mediating the efflux of acetyl-carnitine from mitochondria, thus contributing to the role of CAT in the modulation of the mitochondrial acetyl-CoA pool [12]. As a proof of the crucial role of CAT in metabolism, missense variants of CAT have been linked to Leigh syndrome [13].

**Fig. 1:**
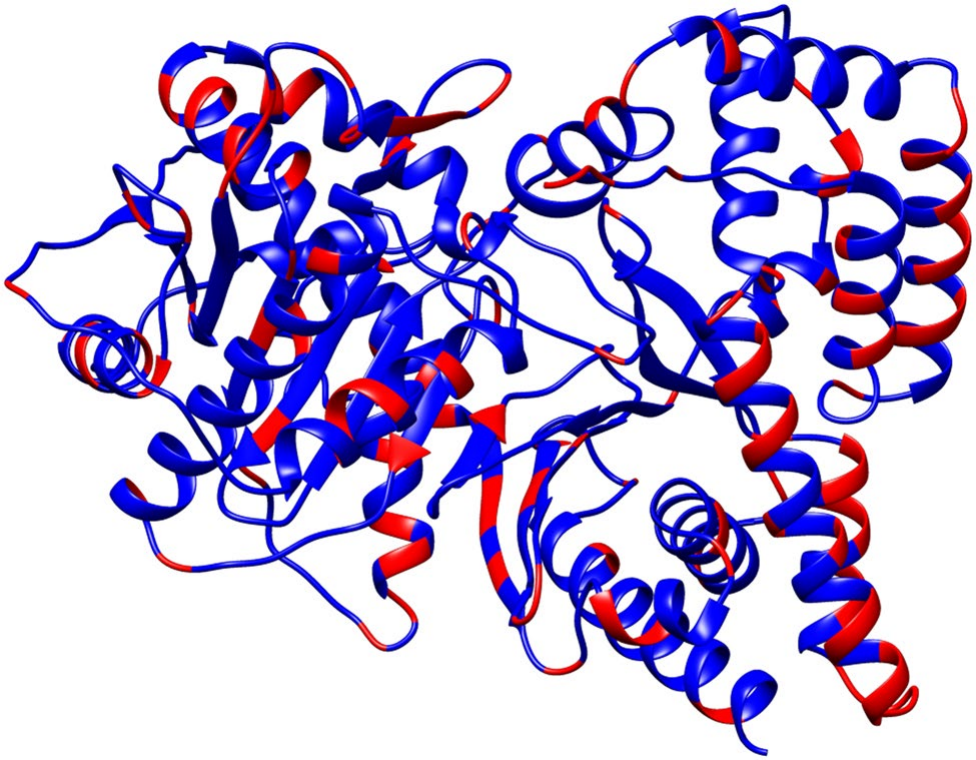
The X-ray crystallographic structure of Human CAT. 3D Structure is shown as a ribbon diagram using UCSF Chimera v.1.14 software [23]. Identical amino acids with pigeon sequence are depicted in blue, whereas non-identical residues are highlighted in red

The study of a human enzyme requires the availability of the human recombinant protein. Indeed, animal proteins are not more considered valuable models for human counterparts [14, 15]. More importantly, the directive 2010/63/EU urges reducing animal experimentation and animal sacrifice by finding novel methodologies that substitute animal models. A functionally active CAT, obtained from pigeon breast muscle, shares only 76% identity with the human CAT (hCAT) (Fig. 1). Therefore, obtaining the recombinant hCAT in a large scale is mandatory for implementing the functional knowledge of this enzyme. Indeed, CAT is interesting for its obvious application to human health.

After the first report on recombinant hCAT protein, which was exclusively used for structural purposes [16], some functional data were described [1]. More recently, some indications for obtaining a recombinant hCAT expression in *E. coli* were published in another paper mainly focused on the link between pathological variants of this enzyme and the Leigh syndrome [13].

However, each cited article lacks some basic information for replicating protein expression. Considering the important role of CAT in cell metabolism and the lack of a defined protocol for producing recombinant hCAT in an active form, we have conceived a novel procedure to fill this gap. In this work, we report a reproducible procedure for the overexpression of hCAT with a higher protein concentration and a much higher enzymatic activity with respect to those previously reported.

## Materials and Methods

### Materials

Acrylamide/Bis-acrylamide (30% solution, 29:1),TEMED (Tetra-methyl Ethylene di-amine), Ammonium persulfate, SDS (Sodium Dodecyl Sulfate), HIS-Select Nickel Affinity Gel (P6611), the Monoclonal Anti-polyHistidine-Peroxidase antibody (A7058), the Carnitine Acetyltransferase pigeon breast muscle, and Carnitine were purchase from Merck; Acetyl-CoA was purchase from Santa Cruz Biotechnology; Agarose, IPTG (Isopropyl-β-D-1-tiogalattopiranoside), DTNB (Ellman's Reagent), Coomassie Brilliant blu R-250 were purchased from AppliChem; nitrocellulose membrane was purchased from GE Healthcare, Life Sciences; pH6EX3 plasmid kindly provided by Brandsch; pET-21a( +) plasmid and *E. coli* Rosetta strain were purchased from Novagen; *E. coli* DH5α strain, restriction endonucleases, Phusion DNA Polymerase, T4 DNA Ligase and specific reagents for cloning were purchased from Thermo scientific; CRAT_OHU15347D_pcDNA 3.1 + /c-(K)-DYK clone was purchased from Genscript; Isolate II PCR and Gel Kit was purchased from Bioline, Meridian Bioscience; QIAprep Spin miniprep was purchased from Qiagen.

### Cloning of hCAT

The 1881 bp cDNA corresponding to the encoding sequence for the Carnitine O-acetyltransferase (NM_000755) was amplified by PCR from the CRAT_OHU15347D_pcDNA 3.1 + /c-(K)-DYK construct using the forward (5′-CGCCATATGTTAGCCTTCGCTGCCAGGACCGTG-3′) and reverse (5′-TAAAGCGGCCGCGAGTTTGGCCCGTGGGTGGCT-3′) primers containing the *NdeI* and *NotI* restriction sites, respectively. The amplified cDNA was then cloned between the *NdeI/NotI* sites in the pET-21a ( +) expression vector carrying a C-terminal 6His tag. Alternatively, the cDNA encoding for the hCAT was amplified from the same template using the forward (5′-CCGGAATTCCATGTTAGCCTTCGCTGCCAGGACCGTG-3′) and reverse (5′-TCGAGTCGACTCAGAGTTTGGCCCGTGGGTGGCT-3′) primers containing the *EcoRI* and *SalI* restriction sites, respectively. The amplified cDNA was then subcloned between the *EcoRI/SalI* sites of the pH6EX3 expression vector carrying an N-terminal 6His tag. PCR reactions contained: 1 unit of Phusion Polymerase, 1X Phusion HF buffer, 0.2 mM dNTP mix, 100 ng of CRAT_OHU15347D_pcDNA 3.1 + /c-(K) -DYK, 400 nM of primers described above and sterile distilled water until 50 µl. An initial denaturation step of 2 min at 98 °C was performed. Then 25 cycles of amplification were performed using the following thermal profile: 30 s denaturation at 98 °C, 30 s annealing at 60 °C, and 1 min extension at 72 °C. An extra extension step of 5 min at 72 °C was performed at the end of the 25 cycles. The amplified PCR products were purified using the purification kit following the supplier's protocol. The purified fragments were digested with *NdeI/NotI* restriction enzymes and ligated into the pET-21 or digested with *EcoRI*/SalI and ligated into pH6EX3. A competent DH5α *E. coli* strain was used for cloning. The miniprep QIAprep kit was used to extract the hCAT constructs from the transformed DH5α. The sequences of the constructs were verified by sending the samples to StarSEQ GmbH (Mainz, Germany).

### Expression of hCAT

Competent *E. coli* Rosetta cells were transformed with 100 ng of pET21_hCAT. The transformed colonies were selected on LB-agar plates added with 100 µg/ml ampicillin and 34 µg/ml chloramphenicol, and then a colony was inoculated overnight in 120 ml of LB, plus antibiotics, at 37 °C under rotary shaking. The day after, the pre-culture was diluted at 1:10 in a fresh medium with specific antibiotics. When the optical density, measured at 600 nm wavelength, reached 0.5, culture was induced with 1 mM of IPTG. After that, the culture was divided into 3 aliquots of 400 ml and grown at 25 °C, 28 °C, and 37 °C for 4 h, respectively. A small aliquot was grown without the inducer as a negative control. After centrifugation at 6000×*g* for 12 min with Beckman Coulter Avanti J-301, pellets were resuspended in 50 ml of a buffer containing 300 mM NaCl, 20 mM Hepes-Tris, pH 7.5, and sonified in an ice bath for 10 min (pulse of 1 s on and 1 s off) at 40 W, using a Vibracell VCX-130 sonifier.

Different IPTG concentrations were tested using a small-scale growth to optimize hCAT production. A pre-culture of 3 ml was diluted in 30 ml fresh medium added with the specific antibiotics. After optical density reached 0.5_600 nm_, the culture was divided into 3 aliquots induced with 0.01, 0.4, and 1 mM of IPTG, respectively. All the aliquots were grown at 25 °C, and 1 ml of each sample was collected at 2 h, 4 h, and 6 h. Each sample was centrifuged at 13,500×*g* for 10 min, using the 5425R Eppendorf centrifuge. The bacterial pellets of small-scale growth were dissolved in 0.5 ml of resuspension buffer described above and subjected to sonication for 5 min (pulse of 1 s on and 1 s off) at 30 W.

Alternatively, competent *E. coli* Rosetta cells were transformed with 100 ng of pH6EX3_hCAT. The transformed colonies were selected on LB-agar plates added with 100 µg/ml ampicillin and 34 µg/ml, and a colony was inoculated overnight in 5 ml of LB, plus antibiotics, at 37 °C under rotary shaking. The day after, the pre-culture was diluted at 1:10 in the fresh medium and used to perform a small-scale growth of 1 ml to test different temperatures (18 °C, 25 °C, 28 °C, and 37 °C), IPTG concentrations (0.01, 0.4, and 1 mM), and time of incubation (2 h, 4 h, and 6 h). All aliquots were centrifuged at 13,500×*g* for 10 min, using the 5425R Eppendorf centrifuge. The bacterial pellet from small-scale growth was dissolved in 0.5 ml of resuspension buffer and sonified for 5 min (pulse of 1 s on and 1 s off) at 30 W.

The total extract from each sample was used to analyze hCAT activity.

Then, soluble and insoluble cell fractions from bacteria grown at 25 °C with 0.01 or 0.4 mM IPTG were separated by centrifugation at 13,500×*g* for 20 min, using the 5425R Eppendorf centrifuge and analyzed by western blotting.

A medium-scale growth of 50 ml under the best condition found for pH6EX3_hCAT (induction with 0.01 mM IPTG, growth at 25 °C, and incubation time of 4 h) was performed. The culture was centrifuged at 4000×*g* for 20 min using 5910R Eppendorf centrifuge, and the bacterial pellet was dissolved in 25 ml of resuspension buffer and sonicated in an ice bath for 10 min (pulse of 1 s on and 1 s off) at 40 W. Bacterial lysate was centrifugated at 13,500×*g* for 20 min, using the 5425R Eppendorf centrifuge. The supernatant was used to perform hCAT purification by nickel affinity chromatography.

### Purification of the hCAT

A 12 ml aliquot of the soluble cell fraction, obtained from Rosetta cells transformed with the pH6EX3_hCAT plasmid and induced with 0.01 mM IPTG and grown at 25 °C, was applied onto a column (h:8 cm, d: 1.7 cm) with 2 ml packed His-Select Nickel Affinity gel. The soluble fraction was incubated with the resin for one hour at room temperature in a fixed angle rotator for tubes. The column was first washed with 40 ml resuspension buffer added with 10 mM Imidazole. Then, 10 ml of resuspension buffer added with 200 mM imidazole were used for eluting purified hCAT. Fractions of 2 ml each were collected. The same protocol was also performed using the soluble cell fraction obtained from Rosetta cells transformed with the plasmid pET21_hCAT, induced with 1 mM IPTG, and grown at 25 °C. The fraction with the greatest amount of purified protein was used to study the hCAT activity.

### Assay of hCAT Activity

Enzymatic activity was determined using a colorimetric assay. hCAT is able to catalyze the following reaction: L-carnitine + Acetyl-CoA ⇌ Acetyl-L-carnitine + CoA-SH. Free CoA thiol group (–SH) can react with Ellman's Reagent (DTNB), forming a yellow product that enables spectrophotometric quantification at a wavelength of 412 nm [17]. The standard assay contains 0.2 mM DTNB, 5 mM L-carnitine, and 0.1 mM acetyl-CoA, 0.1 M EDTA/50 mM Tris–HCl buffer, pH 8.0. The addition of carnitine initiated the reactions, and each measurement was done at the indicated time. The reaction mixture without carnitine was used as a reference for measurement corrections.

### Other Methods

Protein concentration was measured by the method of Lowry [18]. Proteins were separated on 12% polyacrylamide gel by SDS–PAGE, performed according to Laemmli [19], and stained by Coomassie brilliant blue. The western blotting assay was performed using a nitrocellulose membrane and a Monoclonal Anti-polyHistidine–Peroxidase antibody with the dilution of 1:10 000.

## Results and Discussion

### pET21_hCAT Expression and Functional Testing

**Fig. 2:**
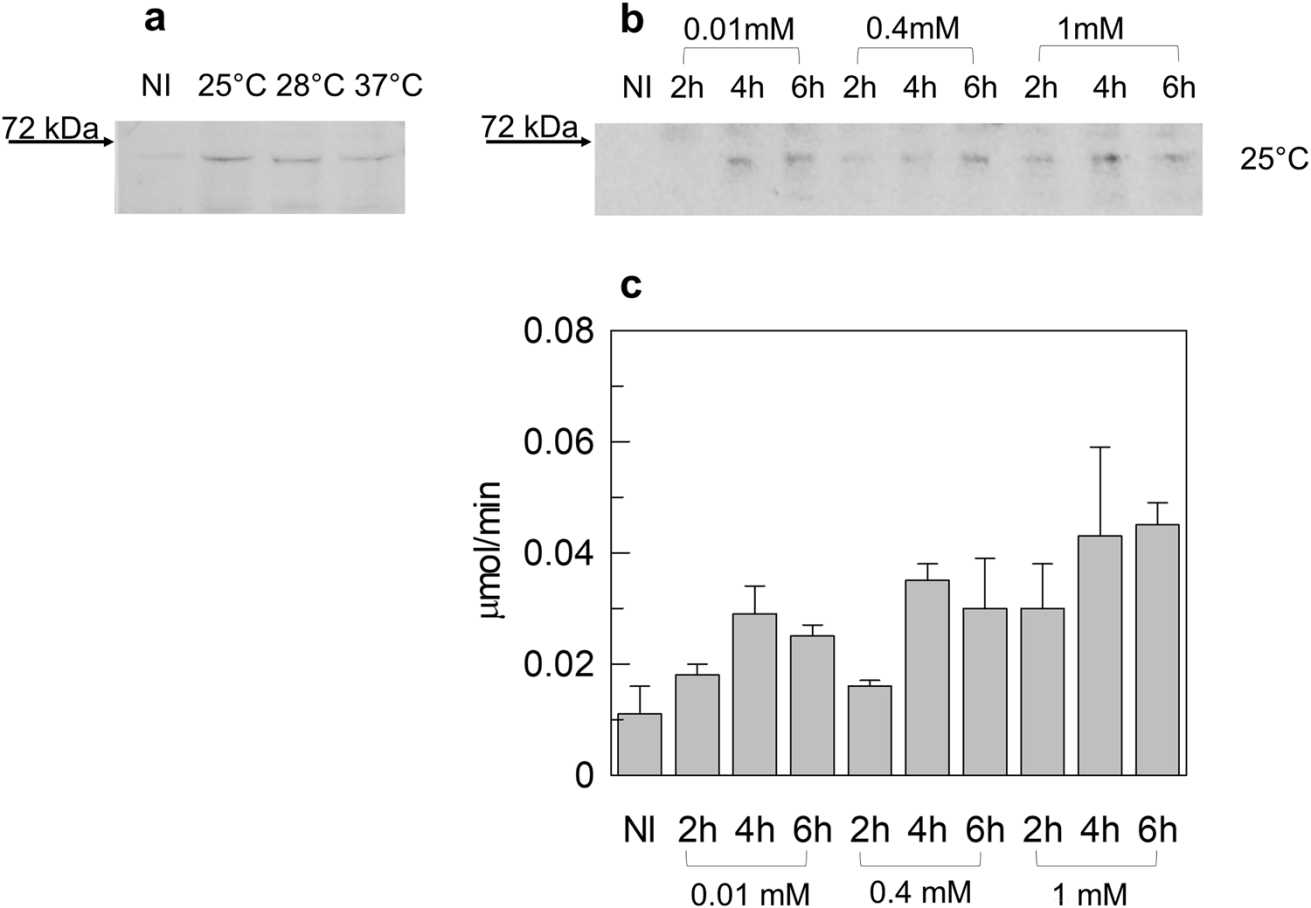
Western Blot analysis and enzymatic activity of pET21_hCAT expressed in *E. coli*. **a** Western Blot of lysates from bacteria cells transformed with pET21_hCAT and immunostained with anti-His tag antibody. Cells were harvested after 4 h of induction with 1 mM IPTG and grown at 25 °C, 28 °C, or 37 °C. **b** Western Blot of Small-scale growth of bacteria cells transformed with pET21_hCAT and cultured at 25 °C. Times of incubation (2 h, 4 h, and 6 h) and concentrations of IPTG (0.01 mM, 0.4 mM, and 1 mM) are indicated. Western blots are representative of at least three independent experiments. **c** Lysates shown in **b** were tested for enzymatic activity as described in the methods section. Time of incubation (2 h, 4 h, and 6 h) and IPTG concentrations (0.01 mM, 0.4 mM, and 1 mM) used for cell growth are indicated. Data represent the means ± SD of at last three independent experiments. *NI* non-induced cell lysate

The cDNA of hCAT was subcloned into the pET21-a ( +) bacterial vector carrying a C-terminal His-Tag, and according to a previous report [13], Rosetta cells were transformed with the pET21_hCAT construct and grown in LB medium until O.D. reached 0.5_600 nm_ [13]. To test the possible influence of temperature on protein expression, cell culture was induced with 1 mM IPTG and then divided into three aliquots grown for 4 h at 25 °C, 28 °C, or 37 °C, respectively. Figure 2a shows the Western Blot analysis of bacterial lysates in the abovementioned conditions. A band with an apparent molecular mass of about 70kDa, which is very close to the theoretical molecular mass of hCAT (70.9 kDa), was detected in all the samples. As expected, the band is virtually absent in the uninduced-cell lysate loaded as a control (NI lane of Fig. 2a). The amount of hCAT decreased by increasing the temperature from 25 to 37 °C. To increase hCAT production, we tested different IPTG concentrations (0.01 mM, 0.4 mM, 1 mM) and growth times (2 h, 4 h, and 6 h). The temperature was set at 25 °C, which was the best condition indicated by the experiment in Fig. 2a. Figure 2b shows that the best expression was obtained by induction with 1 mM IPTG and a growth time of 4 h. The produced hCAT was tested for its enzymatic activity, as described in the methods section. Figure 2c shows that the enzymatic activity increased with increasing IPTG concentration, mostly in agreement with the protein amounts shown by the Western Blot of Fig. 2b. Twenty µl of protein extracts from bacteria cultured with 1 mM IPTG for 4 h or 6 h showed the highest activity, which reached 0.043 ± 0.016 and 0.045 ± 0.004 µmol/min, respectively. The total activity recovered in these samples of 0.5 ml was about 1.1 and 1.13 µmol/min, respectively.

### pH6EX3_hCAT Expression and Purification

**Fig. 3:**
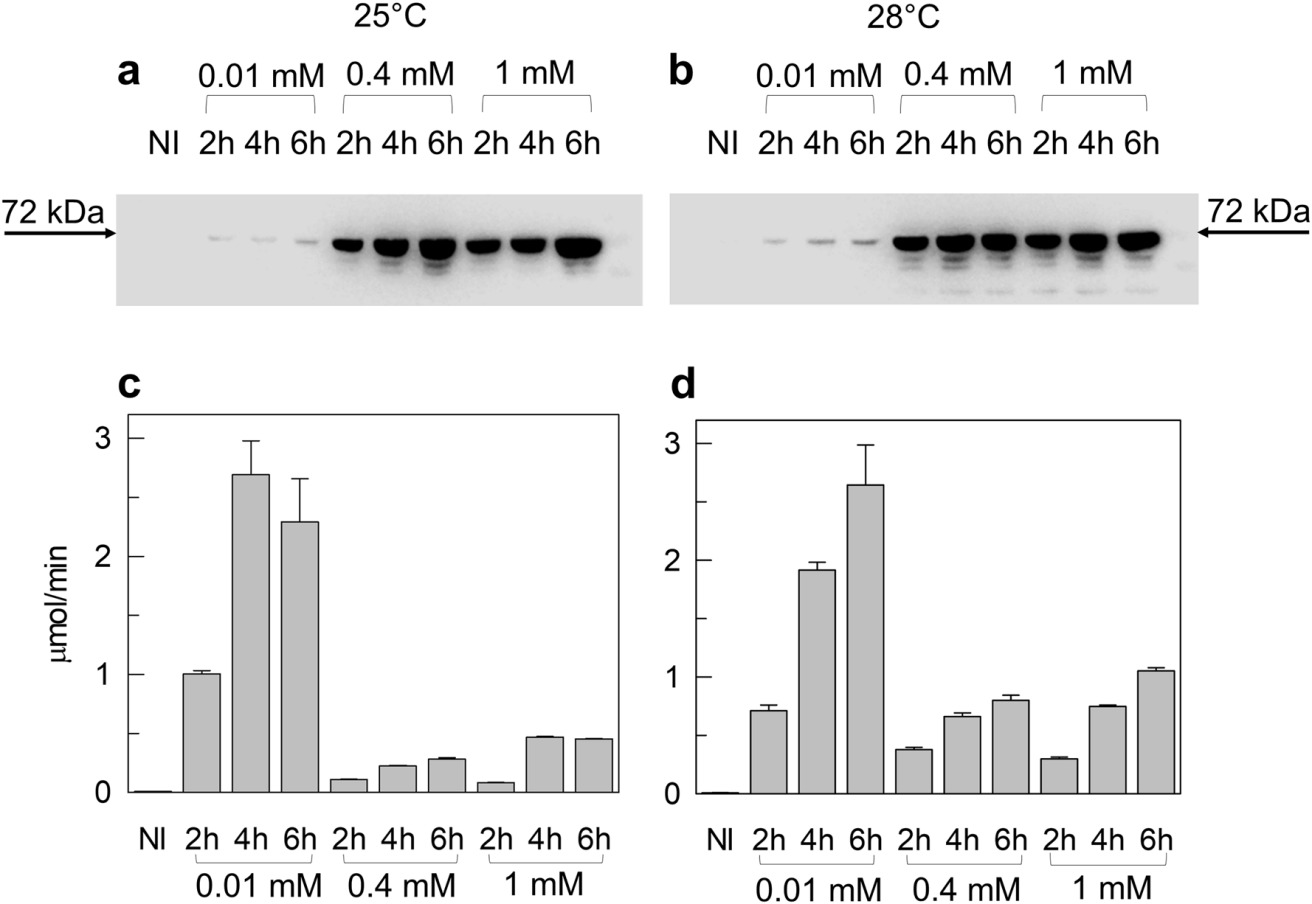
Western Blot analysis and enzymatic activity of pH6EX3_hCAT expressed in Rosetta. Western Blot of Small-scale growth of bacteria cells transformed with pH6EX3_hCAT and cultured at 25 °C (**a**) or 28 °C (**b**). Times of incubation (2 h, 4 h, and 6 h) and concentrations of IPTG (0.01 mM, 0.4 mM, and 1 mM) are indicated. Western blots are representative of at least three independent experiments. **c**–**d** As described in the methods section, the lysates shown in **a** and **b** were tested for enzymatic activity. Time of incubation (2 h, 4 h, and 6 h) and IPTG concentrations (0.01 mM, 0.4 mM, and 1 mM) used for cell growth are indicated. Data represent the means ± SD of at last three independent experiments. *NI* non-induced cell lysate

**Fig. 4:**
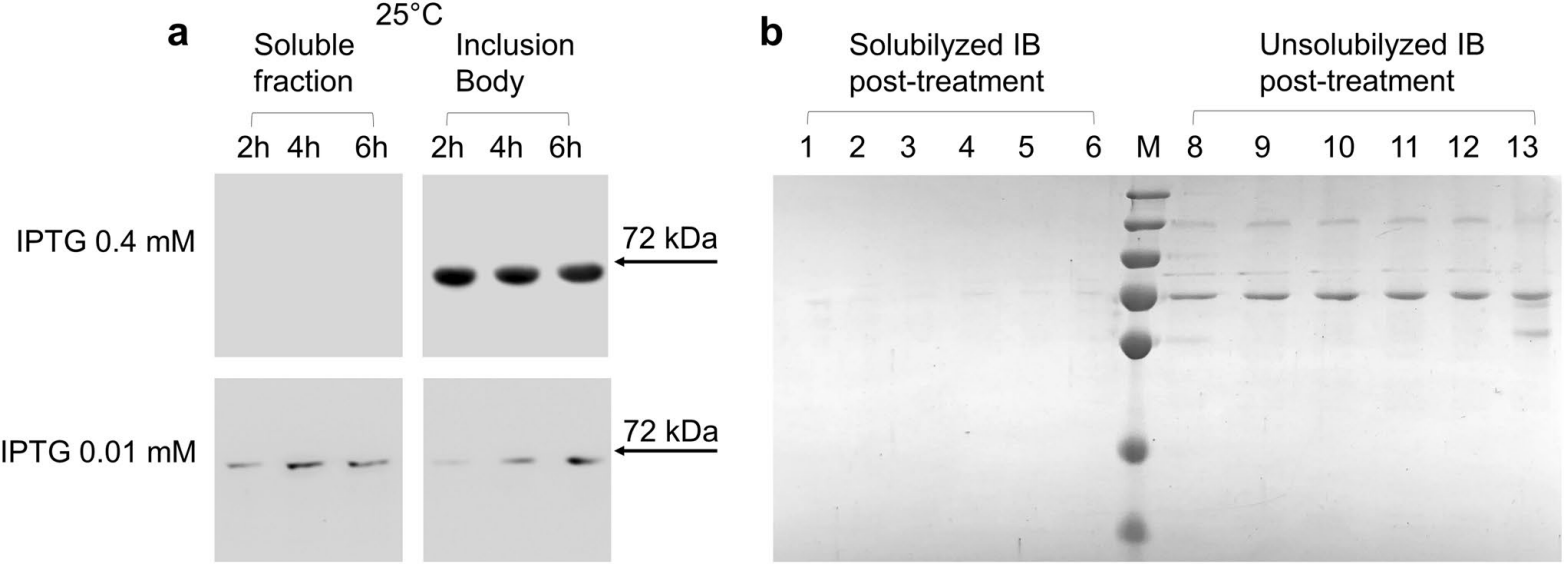
Solubility test of overexpressed CAT. **a** Western blot of soluble fractions or inclusion bodies from lysates of bacterial cells transformed with pH6EX3_hCAT. Cells were induced with 0.01 or 0.4 mM IPTG and grown at 25 °C for 2 h, 4 h, or 6 h, as indicated. **b** SDS-PAGE of inclusion bodies from cells induced with 0.4 mM IPTG and grown at 25 °C for 2 h. After treatment with detergents or DTE (as below described), samples were centrifuged, and supernatants or pellets were subjected to SDS-PAGE analysis and stained with Coomassie blue; lane 1: supernatant of untreated sample; lane 2: supernatant of inclusion body treated with 2 mM DTE; lane 3: supernatant of inclusion body treated with 0,5% Triton-X-100; lane 4: supernatant of inclusion body treated with 2 mM DTE and 0,5% Triton-X-100; lane 5: supernatant of inclusion body treated with 1% C12E8; lane 6: supernatant of inclusion body treated with 2 mM DTE and 1% C12E8; lane M: molecular weight marker (the marker bands from top to bottom: 250 kDa, 130 kDa, 95 kDa, 72 kDa, 55 kDa, 36 kDa, and 28 kDa); lane 8: resuspended pellet from the untreated sample; lane 9: resuspended pellet from inclusion body treated with 2 mM DTE; lane 10: resuspended pellet from inclusion body treated with 0.5% Triton-X-100; lane 11: resuspended pellet from inclusion body treated with 2 mM DTE and 0.5% Triton-X-100; lane 12: resuspended pellet from inclusion body treated with 1% C12E8; lane 13: resuspended pellet from inclusion body fraction treated with 2 mM DTE and 1% C12E8. The experiments are representative of at least three independent experiments

To improve the expression of hCAT, the cDNA was subcloned into the pH6EX3 bacterial vector, which harbors a His-Tag at the N-terminus as in the case of the pQE9 vector used by Govindasamy et al. [1, 20, 21]. The pH6EX3 vector was chosen because it gave high protein expression levels in previous reports [22]. Several concentrations of IPTG (0.01 mM, 0.4 mM, 1 mM) and post-induction growth temperatures (18 °C, 25 °C, 28 °C, and 37 °C) were tested using a small-scale approach. After induction, cells were harvested at 2 h, 4 h, and 6 h. Lysates from the different conditions were subjected to a western blotting assay (Fig. 3a, b, and Fig. S1a, b). The observed main bands showed an apparent molecular mass corresponding to the theoretical one. No bands were detected in the uninduced-cell lysate (NI). However, some bands with a lower molecular mass, probably due to protein degradation, appeared in the lanes corresponding to lysates induced with 0.4 and 1 mM IPTG (Fig. 3a, b, and S1). These bands were most evident at temperatures above 25 °C (Fig. 3a, b, and S1). This suggests that 1 mM IPTG and 30 °C of growth temperature, previously reported for overexpressing hCAT using pQE9 vector [1], were not optimal for pH6EX3_hCAT construct. Figure 3a and b showed that the hCAT expression level using the pH6EX3_hCAT construct was much higher than that obtained using pET21_hCAT (Fig. 2b). A clear dependence of expression on IPTG concentration and time was also observed (Fig. 3a, b). To test if the produced hCAT was active, an equal amount of each sample shown in Fig. 3a and b was used for assaying activity. The highest enzymatic activity was measured in samples induced with low IPTG concentration at 25 °C and 28 °C. Moreover, the measured activity showed a dependence on time at 28 °C while was already maximal after 4 h IPTG incubation at 25 °C. In any condition, the enzyme activity was about two orders of magnitude higher than that of the protein resulting from pET21_hCAT. The maximal activity was 2.69 ± 0.29 µmol/min, corresponding to a total activity of 67.25 µmol/min. Despite a significant expression (see Western Blot in Fig. S1a, b), no or low activity was found in the samples deriving from bacterial growth at 18 °C or 37 °C (Fig. S1c, d). Differently from the pET21_hCAT expression, the highest activity was obtained with the lowest (0.01 mM IPTG) inducer concentration. Under this condition, the protein amount was apparently comparable to that obtained with pET21_hCAT expression. However, this apparent similarity was due to a much shorter exposition of the blotting membrane to avoid the strong saturation of band immunostaining. To clarify the discordance between the apparent expression level and activity, the soluble state of the protein was tested. Samples from lysates of the cells induced with 0.01 or 0.4 mM IPTG were separated into soluble and insoluble fractions by centrifugation and assayed by western blotting. As shown in Fig. 4a, all the hCAT produced by cells induced with 0.4 mM IPTG was in the insoluble fraction, whereas the hCAT from cells induced with 0.01 mM IPTG was enriched in the soluble fraction (Fig. 4a, lower panel). This suggests that hCAT from inclusion bodies is not correctly folded and hence is not functional with a tendency to aggregate. In the attempt to recover the inclusion body protein in a soluble state, the protein was treated with DTE, a thiol reducing agent, or non-ionic detergents such as Triton-X-100 or C_12_E_8_. These compounds have been used individually or in combination with each other. Neither DTE nor detergents were effective in solubilizing the protein (Fig. 4b), highlighting that hCAT produced with higher inducer concentrations as inclusion bodies could not be recovered in a soluble form (Fig. 4b).

**Fig. 5:**
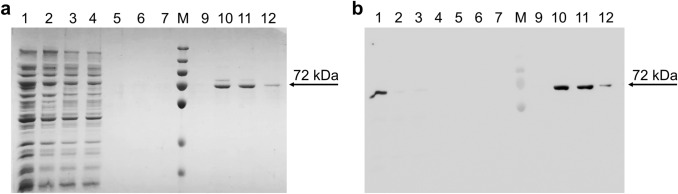
hCAT purification by His-Select Nickel Affinity Chromatography**. a** An aliquot of 15 µl of each fraction of 2 ml obtained were loaded onto polyacrylamide gel and stained with Coomassie blue as described in the methods section. Lane 1: cell lysate; lane 2: passthrough; lane 3: first washing fraction; lane 4: second washing fraction; lane 5: 18th washing fraction; lane 6: 19th washing fraction; lane 7: 20th washing fraction; lane M: molecular weight marker (the marker bands from top to bottom: 250 kDa, 130 kDa, 95 kDa, 72 kDa, 55 kDa, 36 kDa, and 28 kDa); lane 9: first elution fraction; lane 10: second elution fraction; lane 11: third elution fraction; lane 12: last elution fraction. **b** Western Blot of hCAT purification. Lane 1: cell lysate; lane 2: passthrough; lane 3: first washing fraction; lane 4: second washing fraction; lane 5: 18th washing fraction; lane 6: 19th washing fraction; lane 7: 20th washing fraction; *M* molecular weight marker (the marker bands from top to bottom: 250 kDa, 130 kDa, 95 kDa, 72 kDa, 55 kDa, 36 kDa, and 28 kDa); lane 9: first elution fraction; lane 10: second elution fraction; lane 11: third elution fraction; lane 12: last elution fraction; The experiments are representative of at least three independent experiments

According to the above results, the only suitable condition to obtain a high level of hCAT overexpression in the active form consists in using the pH6EX3 construct culturing bacteria with low IPTG concentration at 25 °C to prevent aggregation and degradation. A purification procedure was then attempted on this protein preparation. Cell lysate from 50 ml bacteria cultured as above was centrifuged to remove the insoluble protein fraction. An aliquot of the supernatant was applied onto a Ni^2+^-chelating column. Fractions of 2 ml were collected throughout the entire purification procedure. Figure 5a shows a blue Coomassie-stained gel of this procedure. After loading the soluble fraction of cell lysate to the column (Fig. 5a, lane 1), a washing step was carried out to remove unbound proteins. Indeed, the passthrough and the first two washing fractions (Fig. 5a lanes 2–4) contain many bacterial proteins, except hCAT, which was absent as shown by Western Blot (Fig. 5b lanes 2–4). On the contrary, the last three washing fractions did not contain any proteins, confirming the efficient removal of all unbound proteins (Fig. 5a, b; lanes 5–7). As a control, an aliquot of the soluble fraction used for the purification was loaded in lane 1, which exhibited clear immunostaining of hCAT (Fig. 5b). The recombinant hCAT was eluted, increasing the imidazole buffer concentration. hCAT was mainly recovered in the second, third, and fourth fractions corresponding to lanes 10–12 of Fig. 5a. These lanes showed a single band at 70KDa (Fig. 5a). The same fractions exhibited immunostaining upon western blot analysis, confirming that the purified protein corresponded to hCAT (Fig. 5b, lanes 10–12). From 50 ml of Rosetta cells, transformed with the pH6EX3_hCAT and grown as described above, it was possible to recover 1.56 mg of pure and functional hCAT. Starting from 1 L of culture and after protein concentration, with Centricon Plus-70 Centrifugal Filter, a yield of 30 mg/ml could be reached. This value is higher than (20 mg/ml) previously reported [16].

### Purified hCAT Activity

**Fig. 6:**
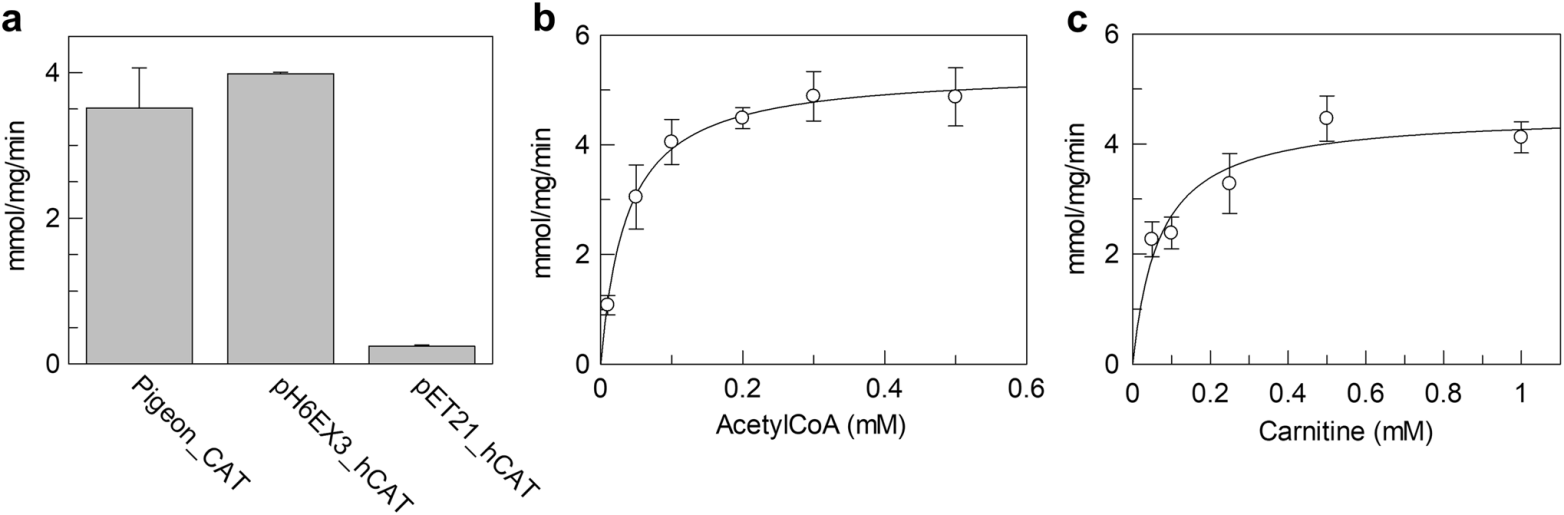
Activity of purified hCAT. **a** The activity of purified obtained using hCAT pH6EX3_hCAT construct compared with hCAT obtained using pET21_hCAT construct or with pigeon CAT. **b–c** Kinetic analysis of purified hCAT; data were plotted according to the Michaelis–Menten equation. The activity was measured as described in the methods section. The data represent the means ± SD of at last three independent experiments

To test the actual functionality of the over-expressed hCAT, the enzyme activity was compared to that of the commercially available CAT extracted from pigeon breast muscle or to the purified pET21_hCAT protein. The pET21_hCAT protein was purified using the same procedure described for pH6EX3_hCAT. Moreover, the activity was also compared to that reported in the literature [1, 13, 16]. As shown by Fig. 6a, the specific activity of the pH6EX3_hCAT protein was nearly coincident with that of pigeon protein, whereas the protein obtained from the pET21_hCAT expression showed a very low specific activity. From these data, it is evident that the specific activity of the protein obtained from the expression of the pH6EX3_hCAT construct is quite similar to the non-human CAT. In addition, the quality and quantity of hCAT obtained by expression of the pH6EX3_hCAT construct are much higher than that obtained using pET21_hCAT construct. Further kinetic analysis was performed to study the dependence of reaction rate on acetyl-CoA and carnitine concentration (Fig. 6b and c). The reaction rate increased by increasing the substrate concentration following a hyperbolic behavior typical of enzyme kinetics. The data were plotted according to the Michaelis–Menten equation; the derived Km for acetyl-CoA and carnitine were 37 ± 3.3 µM and 83 ± 5.5 µM, respectively. These values are similar to those reported previously [1, 13].

Moreover, a kcat value for carnitine of 5678 s^−1^ was measured in this work. It is 147-fold higher than that (38.5 s^−1^) previously reported [1]. This data confirms that the obtained enzyme is virtually fully active and correctly folded, therefore, suitable for functional assays.

### Stability of hCAT

**Fig. 7:**
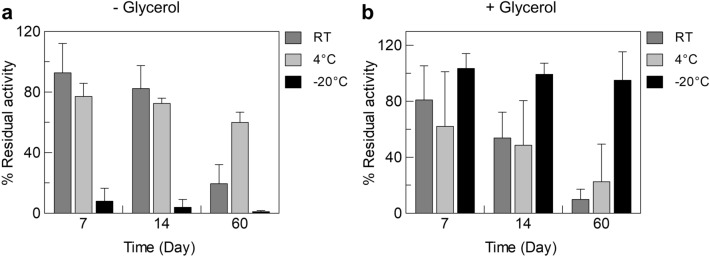
Stability of purified hCAT. Purified hCAT was stored at room temperature, 4 °C, or − 20 °C for indicated times in buffer without glycerol (**a**) or with 50% glycerol (**b**). The activity assay was performed as described in the methods section. The data represent the means ± SD of at last three independent experiments

The stability of purified hCAT was tested over time, up to two months. hCAT was stored at room temperature, at 4 °C or − 20 °C, either in the presence of glycerol or in its absence. Figure 7a shows that after 14 days, hCAT stored at 4 °C or room temperature without glycerol exhibits about 70% or 80% of the initial activity, respectively. After two months, hCAT stored without glycerol at 4 °C shows a residual activity of about 60%. In the presence of glycerol, a faster decrease in activity was observed with time (Fig. 7b). The protein stored without glycerol at − 20 °C is inactive at all the tested times, whereas it is stable after two months if stored in glycerol. The main difference between the two storing conditions is the freezing occurring in the absence of glycerol. Therefore, we can conclude that freezing and thawing alter the protein state in terms of functionality.

## Conclusion

The described data represents the first report on the production of recombinant human CAT, describing the relationship between protein amount, quality, and stability. The obtained hCAT shows a similar specific activity of the commercially available enzyme extracted from a non-human source and is very stable, retaining full activity for at least two months. The produced human enzyme will be very useful for human health applications, particularly for testing biologically active compounds for drug design.

## Supplementary Information

Below is the link to the electronic supplementary material.

Fig. S1 Western Blot analysis and enzymatic activity of pH6EX3_hCAT expressed in Rosetta. Western Blot of Small-scale growth of bacteria cells transformed with pH6EX3_hCAT and cultured at 18°C (a) or 37°C (b). Times of incubation (2h, 4h, and 6h) and concentrations of IPTG (0.01mM, 0.4mM, and 1mM) are indicated. Western blots are representative of at least three independent experiments. (c-d) As described in the methods section, the lysates shown in a and b were tested for enzymatic activity. Time of incubation (2h, 4h, and 6h) and IPTG concentrations (0.01mM, 0.4mM, and 1mM) used for cell growth are indicated. Data represent the means ± SD of at last three independent experiments. NI: non-induced cell lysate (PDF 182 kb)
