# Supplementary material for: Overproduction, Purification, and Stability of the Functionally Active Human Carnitine Acetyl Transferase
Source: Mol Biotechnol. 2022 Jun 21;64(12):1431–40. doi: 10.1007/s12033-022-00522-z (PMC9573857; doi:10.1007/s12033-022-00522-z)

Molecular Biotechnology

Overproduction, Purification and Stability of the Functionally Active Human Carnitine Acetyl Transferase.

Deborah Giudice[1,a], Lara Console[1,2,a], Arduino Arduini[2], Cesare Indiveri[1,2,3]

[1] Department of Biology, Ecology and Earth Sciences (DiBEST), Laboratory of Biochemistry, Molecular Biotechnology and Molecular Biology, University of Calabria, via P. Bucci 4c, 87036 Arcavacata di Rende, Italy;

[2] Unical Cure S.R.L. via P. Bucci 4d, 87036 Arcavacata di Rende, Italy;

[3] CNR Institute of Biomembranes, Bioenergetics and Molecular Biotechnology (IBIOM), National Research Council-CNR, Via Amendola 122/O, 70126 Bari, Italy.

[a] These authors contributed equally to this work.

Corresponding author: Cesare Indiveri, cesare.indiveri@unical.it, University of Calabria, Via Bucci 4C, 87036 Arcavacata di Rende, Italy, ORCID https://orcid.org/0000-0001-9818-6621; tel +390984492939.

Supplementary Fig. 1

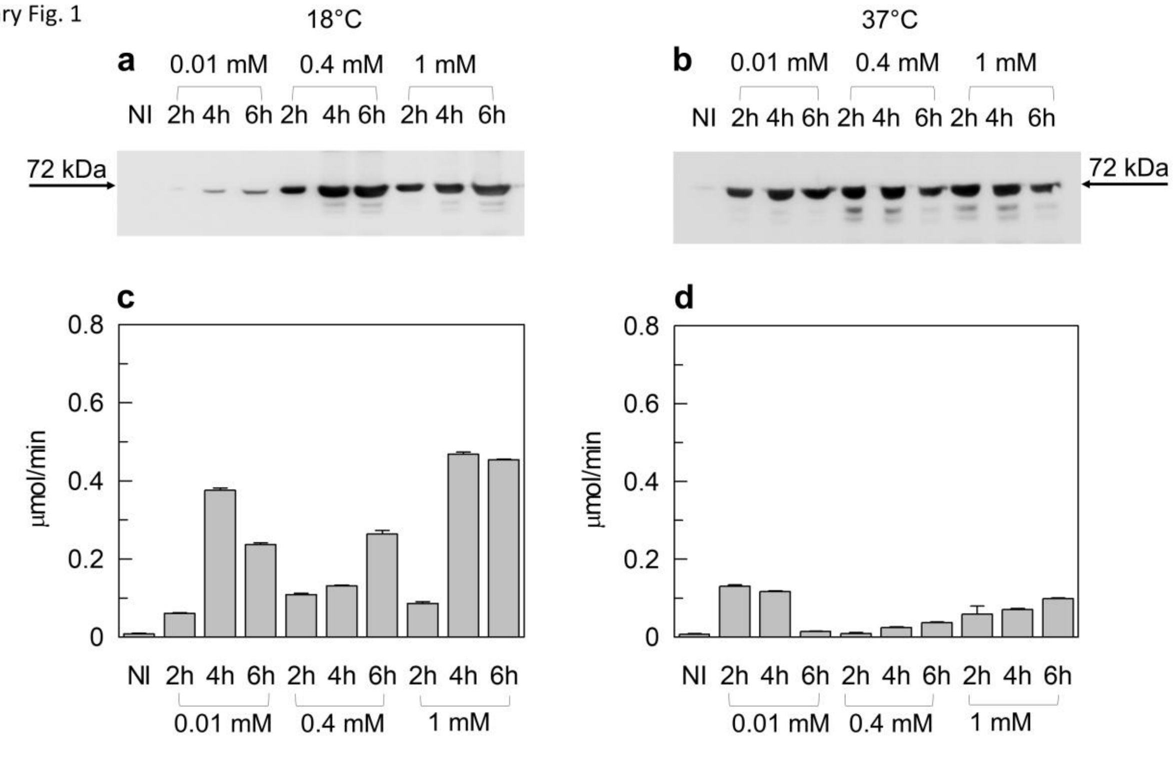

Supplement: Supplementary file 1 — Fig. S1 Western Blot analysis and enzymatic activity of pH6EX3_hCAT expressed in Rosetta. Western Blot of Small-scale growth of bacteria cells transformed with pH6EX3_hCAT and cultured at 18°C (a) or 37°C (b). Times of incubation (2h, 4h, and 6h) and concentrations of IPTG (0.01mM, 0.4mM, and 1mM) are indicated. Western blots are representative of at least three independent experiments. (c-d) As described in the methods section, the lysates shown in a and b were tested for enzymatic activity. Time of incubation (2h, 4h, and 6h) and IPTG concentrations (0.01mM, 0.4mM, and 1mM) used for cell growth are indicated. Data represent the means ± SD of at last three independent experiments. NI: non-induced cell lysate (PDF 182 kb) [file 12033_2022_522_MOESM1_ESM.pdf]
